# The Synergistic Effects of Hyaluronic Acid and Platelet-Rich Plasma for Patellar Chondropathy

**DOI:** 10.3390/biomedicines12010006

**Published:** 2023-12-19

**Authors:** Fábio Ramos Costa, Márcia da Silva Santos, Rubens Andrade Martins, Cláudia Bruno Costa, Paulo César Hamdan, Marcos Britto Da Silva, Gabriel Ohana Marques Azzini, Luyddy Pires, Zartur Menegassi, Gabriel Silva Santos, José Fábio Lana

**Affiliations:** 1 Department of Orthopedics, FC Sports Traumatology Clinic, Salvador 40296-210, Brazil; fabiocosta123@uol.com.br (F.R.C.); clau_costa@uol.com.br (C.B.C.); 2 Metropolitan Union of Education and Culture, Salvador 42700-000, Brazil; marcinha_mairi@hotmail.com; 3 Tiradentes University Center, Maceió 57038-800, Brazil; rubensdeandrade@hotmail.com; 4 Department of Orthopedics, Federal University of Rio de Janeiro (UFRJ), Rio de Janeiro 21941-630, Brazil; hamdanacademico@yahoo.com (P.C.H.); marcosbritto@ufrj.com (M.B.D.S.); zarturmenegassi@ufrj.br (Z.M.); 5 Department of Orthopedics, Brazilian Institute of Regenerative Medicine (BIRM), Indaiatuba 13334-170, Brazil; drgabriel.azzini@gmail.com (G.O.M.A.); luyddypires@gmail.com (L.P.); josefabiolana@gmail.com (J.F.L.); 6 Regenerative Medicine, Orthoregen International Course, Indaiatuba 13334-170, Brazil; 7 Medical School, Max Planck University Center (UniMAX), Indaiatuba 13343-060, Brazil; 8 Clinical Research, Anna Vitória Lana Institute (IAVL), Indaiatuba 13334-170, Brazil

**Keywords:** hyaluronic acid, platelet-rich plasma, patellar chondropathy, regenerative medicine, orthopedics

## Abstract

Musculoskeletal disorders are increasingly prevalent worldwide, causing significant socioeconomic burdens and diminished quality of life. Notably, patellar chondropathy (PC) is among the most widespread conditions affecting joint structures, resulting in profound pain and disability. Hyaluronic acid (HA) and platelet-rich plasma (PRP) have emerged as reliable, effective, and minimally invasive alternatives. Continuous research spanning from laboratory settings to clinical applications demonstrates the numerous advantages of both products. These encompass lubrication, anti-inflammation, and stimulation of cellular behaviors linked to proliferation, differentiation, migration, and the release of essential growth factors. Cumulatively, these benefits support the rejuvenation of bone and cartilaginous tissues, which are otherwise compromised due to the prevailing degenerative and inflammatory responses characteristic of tissue damage. While existing literature delves into the physical, mechanical, and biological facets of these products, as well as their commercial variants and distinct clinical uses, there is limited discussion on their interconnected roles. We explore basic science concepts, product variations, and clinical strategies. This comprehensive examination provides physicians with an alternative insight into the pathophysiology of PC as well as biological mechanisms stimulated by both HA and PRP that contribute to tissue restoration.

## 1. Introduction

**Figure 1:**
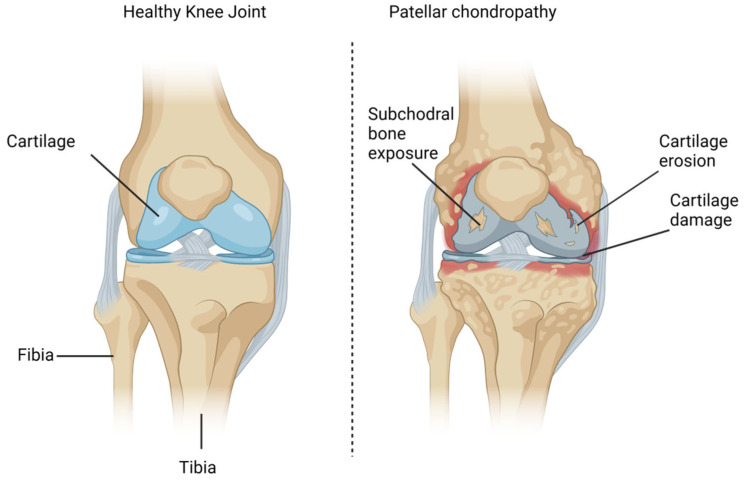
Patellar chondropathy.

Patellar chondropathy (PC), also referred to informally as “runner’s knee”, is an orthopedic condition characterized by visible radiological alterations in patellar cartilage and pain in the anterior aspect of the knee [1]. This condition commonly affects younger individuals, and the initial changes include swelling, edema, and cartilage softening (Figure 1). Notorious factors that contribute to PC are trauma, patellofemoral instability, bony anatomic variations, cartilage vulnerability, abnormal patellar kinematics, or occupational hazards [1].

Although sometimes reversible [1,2], depending on the disease stage (Table 1), PC may progress into patellofemoral osteoarthritis (OA) if left untreated [1]. Significant complications associated with PC include medial meniscus injury in association with impaired knee biomechanics and degenerative alterations (Figure 1), which is highly prevalent in affected patients [3]. Although there is no medical consensus regarding a “gold standard” approach, conservative treatments are usually restricted to rest, immobilization, patella stabilization (braces), orthotics, administration of non-steroidal anti-inflammatory drugs (NSAIDs), and physical rehabilitation exercises [4]. However, physicians should always run a thorough evaluation and consider radiological exams and other clinical findings before making a decision [5,6]. This is of particular importance as the failure of conservative strategies in the advanced stages of PC may eventually require surgical alternatives. This typically includes patellar cartilage excision, shaving, drilling, and distal bony patellar realignment procedures [1].

In end-stage PC and eventual progression into patellofemoral osteoarthritis, patients may require total knee arthroplasty (TKA), which can generate additional problems. On the one hand, TKA mitigates pain and restores gain in range of motion; on the other hand, it may also contribute to quadriceps weakness and reduced functional capacity, propagating physical limitation after TKA procedures [7]. A recent systematic review [8] revealed that patients receiving TKA presented considerable quadriceps weakness, which remained detectable up to 3 months postoperatively.

The emergence of novel treatments employing the use of orthobiologics has provided physicians with feasible alternatives for the management of numerous orthopedic conditions, especially PC [1]. By definition, orthobiologics are organic or synthetic materials that promote enhanced healing of musculoskeletal disorders [9]. Many orthobiologic products have been discussed in the literature, including acellular solutions like hyaluronic acid (HA) and cellular alternatives such as platelet-rich plasma (PRP), injectable platelet-rich fibrin (i-PRF), bone marrow aspirate/bone marrow aspirate concentrate (BMA/BMAC) and adipose tissue derivatives [10,11,12,13,14]. These products can have autologous or allogeneic origins and are known to contain a rich secretome and a wide variety of cells with potent stimulatory effects [15]. These components have shown a satisfactory capacity to modulate the pathophysiology of many disease processes beyond orthopedic conditions, increasing optimism and interest in the field of regenerative medicine [16]. 

HA and PRP (as well as its derivatives), however, have been extensively investigated in the literature for the treatment of musculoskeletal conditions of the knee, especially in terms of degenerative joint diseases [16,17]. Numerous studies [18,19,20,21,22,23,24,25,26,27,28,29] have demonstrated satisfactory outcomes associated with the application of both PRP and HA, although sometimes PRP may or may not prove to be slightly superior to HA. The effects elicited by PRP include cell recruitment, proliferation, differentiation, neovascularization, cytokine secretion, and inflammatory modulation [30]. Similarly, HA attenuates inflammation, induces lubrication, improves biomechanics, cell proliferation, differentiation, and migration, and favors anabolic reactions [13]. Collectively, these responses contribute to pain relief and the regeneration of damaged musculoskeletal structures, including bone, tendon, and cartilage.

The objective of this manuscript is to present the potential applications of PRP and HA as viable orthobiologic tools for the management of PC.

## 2. Methods

The literature was reviewed using PubMed and Google Scholar from April to July 2023 in order to reveal the regenerative medicine potential of hyaluronic acid and platelet-rich plasma. The investigation included the following nomenclature: “patellar chondropathy”, “chondromalacia”, “inflammation”, “orthobiologics”, “hyaluronic acid”, “platelet-rich plasma”. Only full-text articles in English were considered for review.

## 3. Pathophysiology

Patellar chondropathy is also sometimes referred to as “chondromalacia patellae”, which originates from the Greek language and is broken down into two words: “chrondros”, which means cartilage; and “malakia”, meaning softening [1]. Although complex, there are many factors that contribute to the progression of this disease, including direct trauma, patellofemoral instability, bony anatomic variations, cartilage vulnerability, abnormal patellar kinematics, subluxation, or occupational hazards [1,31]. It is worth noting that lifestyle habits may also significantly contribute to general musculoskeletal pathologies affecting locomotor structures, especially in patients with metabolic syndrome (MS) [32,33]. In many ways, PC is quite similar to OA (Figure 1). In fact, it may also be acknowledged as a “pre-osteoarthritic” condition, considering the fact that late-stage PC ultimately leads to the onset of additional complications, such as patellofemoral OA [1,27].

At the anatomical level, many patients suffering from PC usually present a lower lateral patellar tilt angle, lower trochlear depth, and higher sulcus angle due to pathological dysmorphology [3]. The increased trochlear sulcus angle/trochlear depth ratio is, therefore, a helpful tool for physicians as a significant predictor of PC [3]. Another significant indicator of PC is subcutaneous knee fat thickness. According to a study published by Kok et al. [34], the subcutaneous knee fat thickness in obese patients with PC was far greater in comparison to the normal group, therefore establishing a significant correlation between subcutaneous knee fat thickness and grade of PC. Furthermore, female patients analyzed in this study displayed thicker subcutaneous knee fat and more severe grades of PC.

Cartilage damage may occur from either noxious biochemical stimuli or direct mechanical forces [32], promoting alterations (Figure 1) that affect both cartilage and the subchondral bone. The alterations that take place beneath the articular cartilage at the osteochondral junction play a role in the pain and structural progression of the disease, which may affect additional structures and aggravate pathology [35]. When osteochondral integrity is weakened, the boundary between intra-articular and subchondral compartments is lost, thus exposing the subchondral bone and nerves to noxious biochemical and biomechanical stimuli. The diminishing distinction between bone and articular cartilage, coupled with the unavoidable merging of tissue zones at the interface, correlates with the incursion of blood vessels and sensory nerves into the articular cartilage and the forward progression of endochondral ossification [35]. Increased subchondral bone turnover is also intimately connected to these changes at the osteochondral interface [35]; ongoing biomechanical and biochemical strain on articular cartilage further aggravates chondropathy, subsequently generating additional complications such as microfractures, which, in turn, intensify pain [35]. Researchers believe that the various cytokines and growth factors produced in the early stages of the disease may interact with and harm articular cartilage, which also impairs matrix biosynthesis. This process elicits a positive feedback loop mechanism due to repetitive unsuccessful attempts to repair cartilage and bone, eventually progressing into OA [35,36]. In fact, microfractures can serve as channels for the transport of numerous biomolecules [37,38], including inflammatory cytokines. These inflammatory mediators, in turn, may attack vital structures in the joint compartment [39]. A previous animal study [40] outlined the interaction between the subchondral bone and articular cartilage in murine knee joints. After measuring matrix permeability, blood vessel invasion, and joint structure in both surgically induced and age-related OA, the researchers compared observations with the control group. No major alterations in tissue matrix permeability appeared to be connected to the disease in either scenario. However, the pathology provoked significant thinning of the subchondral bone and a rise in blood vessel penetration, which breached the calcified cartilage in both cases. This indicates an increased likelihood of cellular interactions between the subchondral bone and articular cartilage in degenerative pathologies stemming from alterations in the overall joint structure.

## 4. Platelet-Rich Plasma

PRP is a well-known biological material obtained via the centrifugation of peripheral blood with a concentration of 2–5 times above the basal value [16,41,42,43]. It can be prepared either manually or using commercial kits. However, due to the multitude of protocols mentioned in studies, there is a lack of consensus regarding a standardized PRP protocol. This leads to inconsistencies in the composition of PRP products, resulting in the use of diverse terminologies [30,44,45]. 

**Figure 2:**
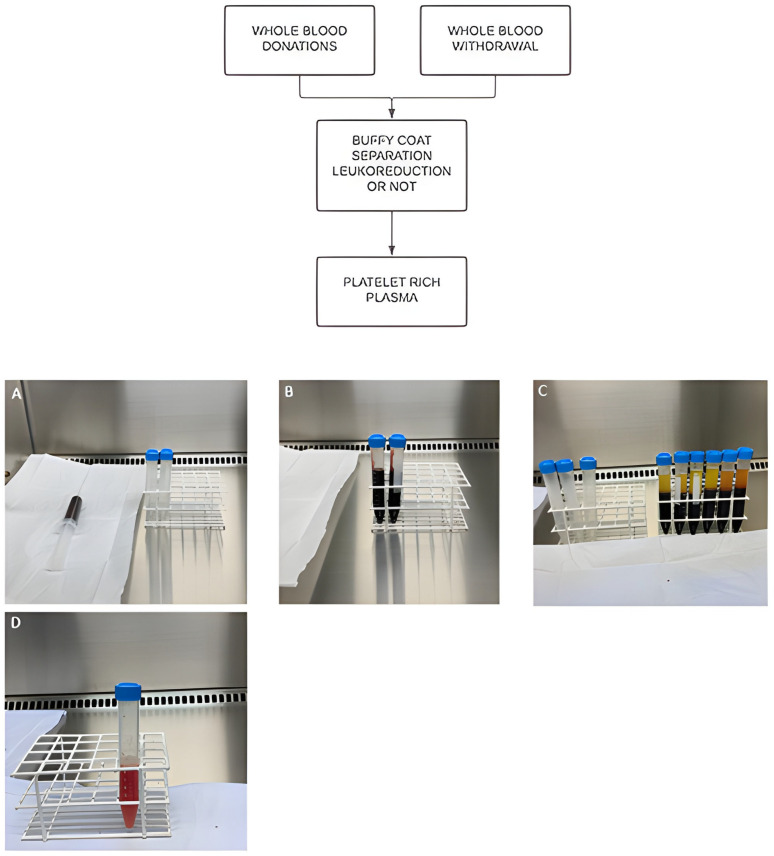
Platelet-rich plasma flowchart. (**A**) collection of the peripheral blood sample using 3.6 mL of the ACD (acid citrate dextrose) anticoagulant; (**B**) adding blood to sterile falcon tubes; (**C**) separation of blood components after first centrifugation (300× *g* for 5 min); (**D**) Resuspended pellets (plasma + buffy coat) without platelet poor plasma give rise to L-PRP (leukocyte-rich PRP) after second centrifugation (700× *g* for 17 min).

Numerous protocols detail the preparation of platelet-rich plasma. Some of them may require one or two centrifugation steps (Figure 2) with precise time and centrifugal force (G) specifications [46]. In any case, PRP is produced by means of density gradients of the cell constituents in blood. Following the initial centrifugation, the densest particles (erythrocytes) are separated from the plasma and settle at the bottom layer of the mixture. Right above the erythrocytes, a thin layer rich in leukocytes (and platelets) forms, making up less than 1% of the total blood; this layer is known as the “buffy coat”. The uppermost layers of this mix also contain platelets and growth factors, being situated immediately above the buffy coat. Plasma (either including or excluding the buffy coat) is then harvested and subjected to a second, final centrifugation to amplify the concentration of platelets [47]. 

The final concentration of constituents in a PRP product can differ based on the commercial kits used or the proficiency of the manual technique, especially if automated processes are not the method of choice [46]. Additionally, the end product can be influenced by individual patient factors. Examples of these factors are age, underlying health conditions, use of certain medications, and blood circulation [48].

Platelet granules possess numerous bioactive compounds. When activated, these compounds are discharged and subsequently trigger the body’s natural healing process [30,49]. Delta granules house substances like magnesium, calcium, adenosine, serotonin, and histamine, which promote clot formation [48]. Alpha granules play a key role in important biological activities like inflammation, blood clotting, immune defense, cell attachment, and cellular proliferation [48]. On the other hand, lambda granules are commonly likened to lysosomes because, just like these organelles, they contain enzymes that break down proteins, lipids, and carbohydrates. As a result, they play a key role in clearing debris and pathogens from damaged tissues [47].

PRP injections can promote rapid neovascularization, enhancing the blood flow and providing essential nutrients to adjacent cells. This is vital for cell rejuvenation and mending of injured tissues [50]. Furthermore, PRP can stimulate many cellular events, including recruitment, division, and differentiation of certain cells, therefore ameliorating the healing of complex wounds and injuries [51]. Indeed, numerous research articles have historically highlighted the advantages of PRP therapy in treating a variety of musculoskeletal conditions, with a particular emphasis on knee pathologies. Recent randomized clinical trials (RCTs), meta-analyses, and systematic reviews [52,53,54,55,56] have reaffirmed that both leukocyte-poor and leukocyte-rich PRP outperform traditional treatments like HA and NSAIDs. In most of these studies, PRP consistently demonstrated more pronounced benefits in reducing pain and enhancing function for those suffering from symptomatic knee OA while ensuring safety and efficacy. Only in one of the most recent RCTs [57] did a singular intra-articular injection of leukocyte-poor PRP combined with HA (Artz or HYAJOINT Plus) prove to be effective and safe for osteoarthritic knees over a 6-month duration. Notable enhancements were observed in measurements like the visual analog scale (VAS) for pain, Western Ontario and McMaster Universities Osteoarthritis (WOMAC) scores, Lequesne indices, and Single Leg Stance (SLS) tests at intervals of 1, 3, and 6 months post-treatment. The application of leukocyte-rich PRP is sometimes frowned upon due to its association with inflammation. However, some researchers point out that in addition to its antimicrobial role, leukocytes can also produce large amounts of VEGF [45], thus being a key contributor to angiogenesis and tissue regeneration [58]. Moreover, neutrophils are also important in these applications as their interaction with platelets promotes a series of reactions that culminate in the production of lipoxin, an anti-inflammatory molecule that limits neutrophil activation and diapedesis [41]. 

Robust studies have been conducted in attempts to decipher the regenerative capacities of platelet concentrates. Historically, the advantages of PRP therapy were largely credited to the rich presence of growth factors and their individual biological impacts (Table 2). Yet, subsequent investigations revealed that PRP also induced remarkable secondary responses, such as the modulation of inflammation [41,59,60,61,62], pro-anabolic stimuli [63], regulation of normal autophagy [64,65], cytokine balance [66,67,68], and anti-catabolic effects [69,70,71]. Moreover, it also plays a direct role in fibrinolytic activities, which are essential for the healing of tissue damage [72]. This holds significant biological importance since the entire fibrinolytic system is vital for attracting mesenchymal stem cells (MSCs), which are crucial for tissue regeneration and repair [30,73]. Finally, an additional positive outcome linked with PRP therapy is the attraction of mononuclear cells. Substances like thrombin and platelet factor 4 (PF4), which are released by platelets, facilitate the gathering of monocytes and drive their transformation into macrophages [74,75,76].

The role of macrophages, in particular, has been much appreciated by researchers due to their adaptability and versatility. Macrophages possess the unique capability to change phenotypes (known as polarization) and even transform into different cell types, such as endothelial cells. This allows them to exhibit varied functions based on the biological signals present in the wound setting [77,78,79]. Macrophages primarily exhibit two main phenotypes: M1 and M2. The M1 phenotype is triggered by microbial elements, taking on a more pro-inflammatory stance linked with wound cleaning or debridement. Conversely, the M2 phenotype typically arises from type II reactions, expressing anti-inflammatory attributes. This is evident through the increased expression of cytokines like IL-4, IL-5, IL-9, and IL-13 [78]. The polarization of macrophages is largely influenced by the concluding phases of wound healing. M1 macrophages induce neutrophil apoptosis, which drives the clearance process [80]. After the phagocytosis of neutrophils, the production of pro-inflammatory cytokines is halted, allowing macrophages to undergo polarization and release TGF-β1. This molecule is fundamental in controlling myofibroblast differentiation, which is essential for wound contraction and closure. Consequently, this facilitates the subsiding of inflammation and initiates the proliferative stage of healing [72].

It is important to mention that while PRP offers numerous advantages, it can also lead to negative outcomes in certain situations. This includes potential risks like infections, the possibility of neurovascular injury, and discomfort at the injection site. These outcomes might be influenced by the expertise (or lack thereof) of the practitioner administering the treatment and the overall health of the patient. Immunocompromised patients or those more susceptible to specific diseases are at a heightened risk of contracting infections at the treated site [81,82]. Therefore, due to variability in the composition of PRP products and the plethora of other orthobiologic products on the market, PRP might not always stand out as the best option for every patient and every musculoskeletal condition [83,84].

## 5. Hyaluronic Acid

**Figure 3:**
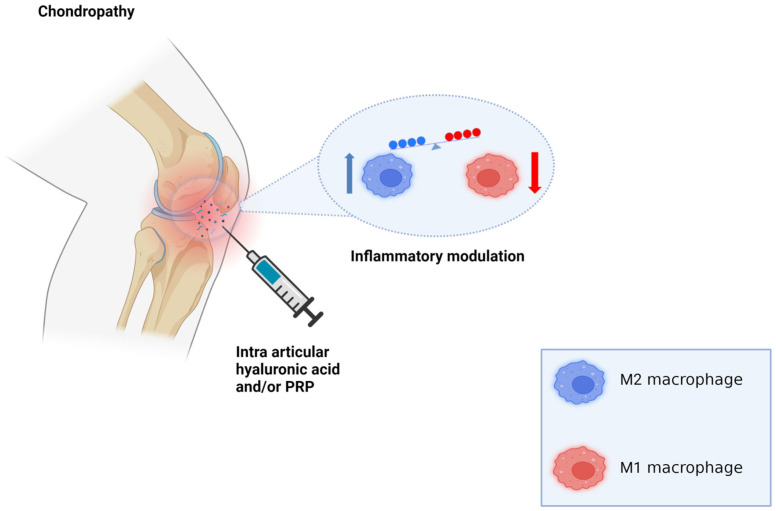
Intra-articular orthobiologic therapy. Intra-articular administration of HA, either alone or in combination with PRP, attenuates exacerbated pro-inflammatory status in patellar chondropathy.

Hyaluronic acid is a negatively charged, nonsulfated glycosaminoglycan that is prevalent in various organ systems [85]. HA products with greater molecular weights typically exhibit anti-inflammatory properties as they control the recruitment of immune cells. Lower molecular weights are known to encourage angiogenesis and tissue restructuring during wound healing; however, they might also show increased pro-inflammatory effects in certain cell types, especially in chondrocytes [86,87]. Low-molecular-weight (LMW) HA, ranging from 500,000 to 730,000 daltons, has a less efficient binding to cell surface receptors. This leads to subdued HA synthesis. In reality, this type of formulation is not very advantageous and is more often linked with inflammation. Tanimoto and colleagues [88] evaluated the impact of the pro-inflammatory cytokines tumor necrosis factor-alpha (TNF-α) and interleukin 1 beta (IL-1β) on rabbit HA-synthetase (HAS) mRNA expression. Under inflammatory conditions, these cytokines elevate HAS mRNA expression, increasing the breakdown and excessive buildup of HA. This, therefore, negatively affects cell activity. Conversely, medium-molecular weight (MMW) HA seems to be the most convenient strategy as it has been shown to facilitate more robust binding. This activates a greater quantity of HA receptors, which in turn boosts the production of endogenous HA. It is also important to highlight that the large molecules typically found in high-molecular-weight (HMW) HA products, for example, might not always display favorable results [89]. While these large molecules still connect to HA receptors, their expansive domains may restrict the availability of unoccupied binding sites on the cell surface. This suggests a potentially reduced efficiency in stimulating HA synthesis [89]. Interestingly, however, one study [90] revealed that HMW HA (>1250 kDa) inhibits pro-inflammatory responses in lipopolysaccharide-mediated macrophage activation. Additionally, it promotes anti-inflammatory responses according to concentration, especially via the expression of genes associated with the M2 macrophage phenotype [90]. Another study [91] showed that intermediate-sized hyaluronan fragments may also play an immunomodulatory role as they interact with toll-like receptor 4 (TLR4) and orchestrate macrophage polarization to an M2-like phenotype (Figure 3).

In any case, HA is still a critical component of articular cartilage, where it forms a protective layer around chondrocytes. It serves as a lubricant for tendons and joints, diminishing the breakdown of extracellular matrix (ECM) by inhibiting the synthesis of matrix metalloproteinase (MMP). Its anti-inflammatory properties reduce the effects of TNF-α and interleukin-1 (IL-1), both primary pro-inflammatory agents. As an independent entity, HA proves to be a powerful tool in managing degenerative conditions of the knee, in particular. In fact, its advantages in orthopedic scenarios have been widely recognized in academic circles for years. More recent systematic reviews reaffirm that intra-articular (IA) uses of HA are not only safe but also cost-effective. They reduce pain and enhance knee functionality when assessed against conventional treatments like NSAIDs, corticosteroids, and other painkillers in general [92,93].

Intra-articular hyaluronic acid (IA-HA) is viewed as a minimally invasive therapeutic approach, and there have been no major systemic side effects documented [94]. This method has demonstrated positive outcomes in laboratory settings. Not only has IA-HA been found to decrease chondrocyte cell death, but it also boosts their growth [95]. For human use, it is best to rely on formulations of medium- (800,000–2,000,000 daltons) to high-molecular-weight HA, as this closely mirrors the conditions and biological attributes of HA naturally generated in the body. Additionally, it is crucial to employ HA sourced from biological synthesis to prevent unwanted adverse reactions [96].

The molecular mechanism at play is associated with the innate ability of HA to bind to a cluster of differentiation 44 (CD44) receptors. By doing so, it inhibits the expression of IL-1β, leading to a decreased production of MMPs 1, 2, 3, 9, and 13 [97,98,99], circumventing the action of degradative enzymes in the musculoskeletal framework [100]. Upon connecting to its receptor, HA activates internal signaling routes related to its own proliferation, differentiation, migration, and breakdown [101]. CD44 is the most extensively researched HA receptor due to its presence in almost all human cell types. The affinity between CD44 and HA is vital in determining HA’s capacity as a signaling molecule. Nonetheless, this relationship also hinges on factors such as HA concentration and molecular weight, along with the glycosylation of external domains and serine phosphorylation [102]. CD44 aggregates with HMW HA polymers, facilitating interactions with growth factors, ECM proteins, MMPs, and cytokines [103]. The other notable HA surface receptor is CD168. It is present in various cells and governs migration through its interaction with skeletal proteins, even more so during the healing process [102].

HA conveys its protective benefits through two clear phases. The initial phase, referred to as the mechanical stage, sees the synovial fluid being replaced with denser concentrations of HA, resulting in increased viscosity [17]. Furthermore, this facilitates the enhancement and recovery of the lubrication and shock-absorbing attributes of the synovial fluid. It forms a protective layer around pain receptors, diminishing pain signals [104]. The subsequent and concluding phase is commonly referred to as the pharmacological stage, in which the biosynthesis of native HA and ECM components occurs [105]. This decreases the depletion of proteoglycans in the cartilage and avoids chondrocyte apoptosis [106]. It also reduces the activity of inflammatory cells, thereby minimizing HA breakdown and the generation of pain-inducing agents [17].

Additional benefits of HA go beyond the traditional lubricating and shock-absorbing properties. According to a recent systematic review [107], HA also displays senomorphic properties. Senomorphics refers to a group of therapeutic agents capable of manipulating the function and morphology of senescent cells to a similar profile seen in young cells or at least delaying the progression of senescence [107]. In other words, HA can control the production of senescence-associated secretory phenotype (SASP) and other substances, such as cytokines and MMPs, which are involved in cellular damage due to aging [107]. HA’s senotherapeutic potential is mainly attributed to the upregulation of sirtuin 1 and its reductive effect on oxidative stress [108], marked by a particular reduction in MMP 13, specifically [109]. Moreover, HA can also improve mitochondrial activity, which is essential for cellular homeostasis and longevity. 

## 6. Discussion

In clinical trials, Synvisc (6000 kilodaltons) and Hyalgan (500,000–730,000 daltons) were the most commonly utilized HA products because of their safety, effectiveness, and enduring benefits, even though they require intra-articular injections [102,110]. Specifically, Hyalgan has demonstrated its ability to boost the survival and growth of human chondrocytes when subjected to reactive oxygen species (ROS) [111]. In recent times, a novel product composed of a blend of HA and lactose-modified chitosan (Chitlac^®^) has demonstrated encouraging outcomes in amplifying the anti-inflammatory response and therapeutic efficacy of HA for knee pathologies. Both in vitro and in vivo research have indicated a notable enhancement in cartilage regeneration following the application of this derivative in experimentally triggered knee OA, for example [112,113]. A recent 2021 study further illuminated its benefits. The combination of HA and Chitlac^®^ notably reduces the cytotoxicity of the triamcinolone acetonide-hydroxypropyl-β-cyclodextrin (TA-CD) drug in human chondrocyte cultures. It also maintains its anti-inflammatory properties, underscoring once more the chondroprotective function of HA in the management of inflammatory knee conditions [114]. However, in the face of more advanced and severe stages of joint degeneration, HA by itself might not be enough, creating the need for further treatments like autologous chondrocyte implantation or even PRP injections. Three-dimensional scaffolds made from biodegradable and biocompatible HA-based polymers, like Hyaff-11^®^, have been effectively used in the past for cultivating human chondrocyte cultures [115]. After the chondrocytes are implanted, the newly regenerated tissue matures and becomes hyaline tissue, as opposed to forming fibrous cartilage [115].

An RCT [116] evaluated the clinical outcomes of PRP and HA both individually and in combination for treating mild to moderate knee OA in 105 patients. The participants were randomly assigned to HA, PRP, or a combination of HA+PRP groups. They were administered three intra-articular knee injections of their designated substance, with 2-week gaps between each injection. The clinical results were assessed using the Western Ontario and McMaster Universities Arthritis Index (WOMAC) and the Visual Analogue Scale (VAS) questionnaires at the start and after intervals of 1, 3, 6, and 12 months. The combination of HA and leukocyte-rich PRP was determined to have a significant positive impact on clinical outcomes, with noticeable improvements in physical function and pain reduction observed within the first 30 days post-treatment. These findings might be linked to the ability of HA to offer a functional matrix that possesses supportive scaffold properties, which can enhance cartilage biomechanics and promote tissue repair [117]. A recent study published by Lana et al. [12] offers a detailed description of the “platelet-rich plasma power mix gel”, a robust orthobiologic product that combines PRP, PRF, and HA for enhanced effects.

There are quite a few factors that must be taken into consideration before designing a specific treatment strategy for patients. First and foremost, a practitioner must consider the safety, efficacy, and practicality of orthobiologic preparation, no matter what they are. For example, there are certain processing hurdles associated with viable PRP processing methods. If the manual alternative is chosen, the orthopedic practitioner will need adequate room, time, equipment, and qualified personnel in order to adequately collect and process blood to avoid sample contamination [118], therefore raising costs. The patient’s lifestyle (i.e., diet, drug consumption, stress) and overall metabolic health status are also important because these individual factors influence PRP efficacy and treatment outcome [32,119]. Commercially available PRP kits are not faring any better [120], as quality and quantity may also differ for various reasons. Moreover, they are also comparably expensive and, therefore, limit their widespread use across a broader population [121]. HA application, on the other hand, is faster and does not require any sort of processing, as the vials are readily available for easy aspiration and injection.

In regards to cost-effectiveness and comparison with other known treatments for orthopedic conditions, a recently published study has compared IA administration of HA versus PRP for the treatment of symptomatic knee osteoarthritis [122]. In their research, Samuelson and team reported that the cost per quality-adjusted life-year (QALY) for a series of PRP injections stood at USD 8635.23/QALY, in contrast to HA injections, which cost USD 5331.75/QALY. PRP, however, demonstrated a significantly higher effectiveness at the one-year mark. Rosen et al. [123] sought to compare IA-HA with conservative interventions such as physical therapy, orthosis, NSAIDs, and other painkillers for early to mid-stage knee OA. In this specific scenario, their findings indicated that HMW HA surpassed LMW HA and physical therapy, offering a more cost-efficient solution while delivering better results. When compared to orthosis and NSAIDs/analgesics, HMW HA proved to be cost-effective.

## 7. Author’s Note

It is once again important to emphasize that precise control over the patient’s metabolic health is of great value. Individuals affected by MS are known to have complications regarding tissue repair [32,124]. MS is a cluster of metabolic dysregulations that lead to a state of chronic low-grade systemic inflammation (meta-inflammation) [125]. These dysregulations are commonly referred to as “The Deadly Quartet”, encompassing insulin resistance, dyslipidemia, visceral obesity, and hypertension [32]. In the context of PC, MS itself does not significantly influence cartilage regeneration; however, it does push cartilage homeostasis towards deterioration. Of the various factors associated with metabolic dysregulation, hypertriglyceridemia notably exerts a distinct impact on cartilage metabolism [124]. The state of chronic low-grade systemic inflammation affects many organs and anatomical structures. The exact metabolic pathways through which obesity, in particular, contributes to structural damage of joints do not seem to be fully elucidated. However, it is believed that the elevated adipokine expression from adipose tissue elicits direct and downstream effects which lead to the destruction and remodeling of the joint as whole [126].

Perhaps the most simple and cost-effective approach to mitigating PC progression is to simply adjust lifestyle habits. This is especially relevant since many risk factors associated with MS are amendable. For example, avoiding diets high in saturated fats and refined carbohydrates, combined with regular moderate exercise in order to reduce weight and the biomechanical burden on the knee can reduce the detrimental impacts brought on by MS. The ingestion of nutraceuticals such as red-yeast rice, berberine, curcumin as well as vitamin D, for instance, are known to improve lipid handling by the liver and ameliorate insulin resistance [127]. The Mediterranean diet, in particular, targets MS components due to its elevated quantities of dietary fiber, omega 3 and 9 fatty acids, complex carbohydrates, antioxidants, minerals, vitamins, and bioactive substances, including polyphenols [128]. These nutraceutical compounds counteract MS by regulating the gut microbiome and gastrointestinal function and shielding cellular components against oxidative stress and inflammation [128]. In terms of physical activity, according to the literature, it appears that any aerobic training program (brisk walking, cycling, swimming, jogging, or dancing) of 16 weeks with a frequency of three times per week can improve cardiorespiratory fitness in sedentary individuals with MS [129]. In order to manage knee pathologies, neuromuscular exercise and balance training seem to be the most effective options for improving proprioception, sensorimotor control, and functional stability. Among these, aquatic exercise is gaining popularity due to its lower incidence of side effects compared to other forms of exercise training [130].

Nevertheless, it is important to note that with weight reduction, there may also be relative loss of muscle mass and strength over time as a result of protein deficiency in these patients, which may lead to the recurrence of PC symptoms [131]. Physicians should manage musculoskeletal diseases using both conservative therapies and surgical options on a case-by-case basis, as patients may experience similar conditions but respond differently to treatments. Rather than focusing solely on orthobiologic therapy, practitioners must be even more attentive to a patient’s metabolic health status. Previous studies have depicted a positive correlation between fat tissue volume and PC, including degree of severity [132,133]. Overall, the association of HA and PRP (Figure 3) may promote better clinical outcomes during the early stages of the disease, such as grade I or II (Table 1), where the pathological alterations are moderate and still amendable with less invasive techniques [1]. 

## 8. Conclusions

Hyaluronic acid is a crucial biological compound naturally present in many tissues. It plays key physiological roles in supporting musculoskeletal integrity, especially in regard to debilitating orthopedic diseases like patellar chondropathy. Intra-articular injections of hyaluronic acid represent a minimally invasive approach with proven efficacy and safety. This treatment option offers numerous improvements that target inflammation, lubrication, biomechanics, cell growth, differentiation, migration, and protein biosynthesis. 

PRP products obtained by whole blood centrifugation are another viable orthobiologic tool aiming at an increased concentration of platelets, surpassing the baseline level. This tool fosters rapid growth in bone and soft tissues with minimal adverse reactions. Autologous PRP therapy has consistently demonstrated promising clinical outcomes in stimulating and augmenting the healing of various tissue injuries, just like hyaluronic acid. The effectiveness of this alternative treatment is attributed to the release of a diverse set of bioactive molecules and its capacity to modulate the inflammatory cascade, which reinforces tissue regeneration.

While both of these orthobiologic tools have their own fair share of advantages and drawbacks, they still promote similar positive effects in degenerative joint conditions like patellar chondropathy. Success rates may be higher in the early stages of disease, where the pathological alterations are moderate. Also, the management of other comorbidities, such as metabolic syndrome, is also a wise strategy that may improve clinical outcomes. Although there is an increasing array of new orthobiologic products intended for degenerative joint conditions affecting the patella, further research is still needed in order to better understand the factors that contribute to musculoskeletal tissue restoration.

## Figures and Tables

**Table 1:** Outerbridge classification scale for patellar chondropathy.

Grade	Radiological Observations
Grade 0 (normal)	No radiological findings
Grade I	Softening and swelling, edema
Grade II	Fragmentation and fissuring in an area of about 1.27 cm^2^ (half an inch) in diameter
Grade III	Acute fragmentation and fissuring in an area greater than 1.27 cm^2^ (half an inch) in diameter
Grade IV	Severe cartilage denudation and erosion down to the subchondral bone compartment

**Table 2:** Typical growth factors found in PRP.

Growth Factor	Abbreviation	Biological Function
Insulin-like growth factor	IGF	Promotes cell growth and differentiation, stimulates collagen synthesis and cell recruitment from bone, endothelium, epithelium and other tissues.
Vascular endothelial growth factor	VEGF	Stimulates angiogenesis, chemotaxis of macrophages and neutrophils, migration and mitosis of endothelial cells, and increases permeability of blood vessels.
Hepatocyte growth factor	HGF	HGF is secreted by mesenchymal cells and stimulates mitogenesis, cell motility, and matrix invasion.
Fibroblast growth factor	FGF	Regulates cellular proliferation, survival, migration, and differentiation.
Epidermal growth factor	EGF	Sustains proliferation and differentiation of epithelial cells, promotes secretion of cytokines by mesenchymal and epithelial cells.
Transforming growth factor-β	TGF-β	Increases synthesis of collagen type 1, stimulates angionesis and immune cell chemotaxis, and inhibits osteoclast formation and bone resorption.
Platelet-derived growth factor	PDGF	Increases expression of collagen, proliferation of bone cells, fibroblast chemotaxis and proliferative activity, and induces macrophage activation.
